# Intestinal Obstruction Caused by Ileocolic and Colocolic Intussusception in an Adult Patient with Cecal Lipoma

**DOI:** 10.1155/2016/3519606

**Published:** 2016-12-04

**Authors:** Tiziana Casiraghi, Alessandro Masetto, Massimo Beltramo, Mauro Girlando, Camillo Di Bella

**Affiliations:** ^1^Azienda Socio-Sanitaria di Vimercate, Presidio di Carate, Via Mosè Bianchi 9, 20841 Carate Brianza, Italy; ^2^Azienda Socio-Sanitaria di Vimercate, Presidio di Vimercate, Via Santi Cosma e Damiano 10, 20871 Vimercate, Italy; ^3^Azienda Ospedaliera di Desio e Vimercate, Presidio di Carate, Via Mosè Bianchi 9, 20841 Carate Brianza, Italy

## Abstract

*Introduction*. Intussusception is a rare clinical entity in adults (<1% of intestinal obstructions). Colonic intussusception is even rarer, particularly when caused by lipomas.* Case Presentation*. A 47-year-old woman presented to our emergency department complaining of abdominal pain with vomiting and diarrhoea. X-ray and CT showed bowel obstruction due to ileocolonic and colocolonic intussusception; a giant colonic lipoma (9 × 4 × 4 cm) was recognizable immediately distally to the splenic flexure of the colon. The patient underwent emergency laparotomy and right hemicolectomy. Assessment of the resected specimen confirmed the diagnosis of giant colonic polypoid lesion near to the ileocecal valve, causing a 12 cm long intussusception with moderate ischemic damage.* Conclusion*. Colonic obstruction due to intussusception caused by lipomas is a very rare condition that needs urgent treatment. CT is the radiologic modality of choice for diagnosis (sensitivity 80%, specificity near 100%); since the majority of colonic intussusceptions are caused by primary adenocarcinoma, if the etiology is uncertain, the lesion must be interpreted as malignant and extensive resection is recommended. At present, surgery is the treatment of choice and determines an excellent outcome.

## 1. Introduction

Lipoma of the gastrointestinal tract is a rare condition described for the first time in 1757 by Baurer and reported in only 0.2%–4.4% of large autopsy series since 1955 [1].

Intussusception was first described by Barbette of Amsterdam in 1674 [2]. It is relatively frequent in children but rare in adults, representing 5% of all bowel intussusceptions and 1% of all bowel obstruction [2, 3].

Colic intussusception is even rarer, above all when caused by lipomas: thirty-seven definite cases have been reported in the English-language literature over the past 45 years [4].

## 2. Case Report

A 47-year-old woman presented to our emergency department with abdominal pain associated to vomiting and diarrhoea for one week. She was known for a suspicious history of Crohn.

**Figure 1:**
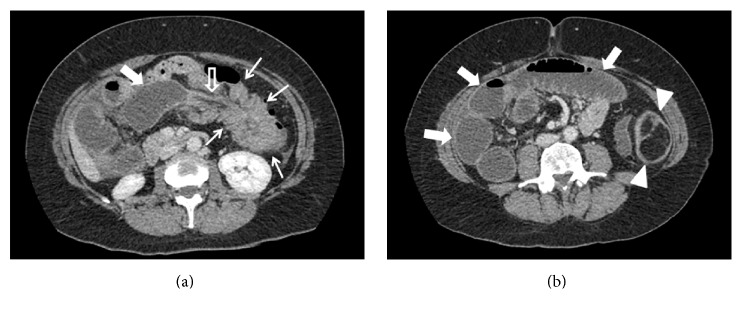
Contrast-enhanced CT, portal venous phase, axial images. In (a) the last ileal loop is dilated (full arrow); a part of the right colon and its mesentery (empty arrow) are recognizable inside the left colon (thin arrows). In (b) the section is at a lower level; on the left side, the colonic lipoma (arrowheads) is recognizable as a lobulated lesion with fat-equivalent density and with inner septa. On the right side notice dilated small bowel loops (full arrows) and absence of the right colon.

**Figure 2:**
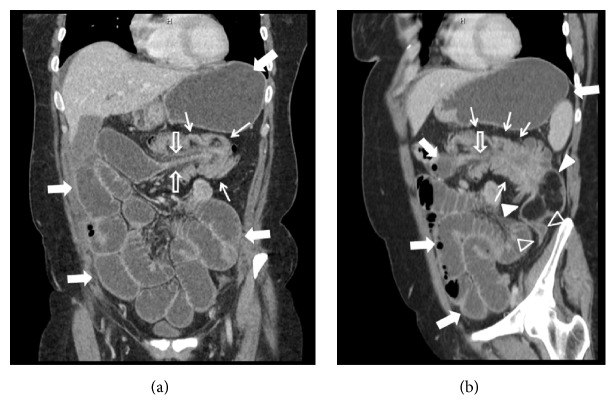
Contrast-enhanced CT, portal venous phase, multiplanar reconstructions (coronal plane, (a), and paracoronal plane, (b)). The same features described in (a) can be appreciated: the dilated stomach and small bowel loops (full arrows), the right colon, intussusceptum (empty arrows), the left colon, intussuscipiens (thin arrows), the colonic lipoma (full arrowheads), and the collapsed left colon distal to the lipoma (empty arrowheads).

**Figure 3:**
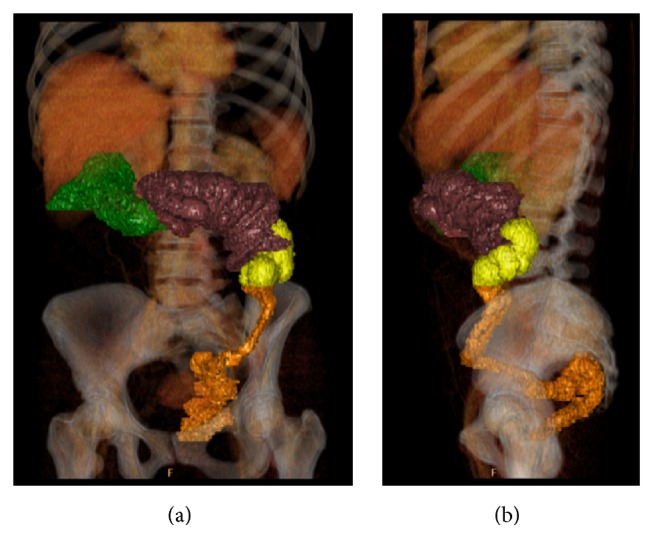
Contrast-enhanced CT, portal venous phase, 3D volume rendering reconstructions. The anatomical structures of interest were segmented for a panoramic view: the dilated last ileal loop (green), the intussuscepted colonic segments (purple-brown), the lipoma (yellow), the collapsed distal third of the left colon, and the sigmoid and rectum (orange). The skeletal structures and the parenchymatous organs were left in transparency.

In the emergency department laboratory tests showed PCR 5.89 mg/dl and Hb 10.3 g/dl; an X-ray showed signs of bowel subocclusion. The patient was admitted to the hospital and a CT scan revealed the bowel obstruction with ileocolonic and colocolonic intussusception as far as splenic flexure of the colon; on the left side there was a formation characterised by fat-equivalent density and intralesional septa. CT findings were suggestive of giant lipoma (9 × 4 × 4 cm). Moderate free fluid was also present (Figures 1, 2, and 3).

The patient underwent emergency laparotomy. Surgical exploration confirmed the colocolonic intussusception; the last ileal loops migrated in the colon, too. The condition appeared to be due to an intraluminal colonic polypoid lesion, appreciable at the level of the splenic flexure at palpation.

Conservative treatment was not possible due to ischemia of involved segments, and right hemicolectomy was performed. The resection was extended from the last ileal loop to the right colon as far as the big polypoid lesion. An ileotransverse colon manual anastomosis was performed.

The postoperative course was uneventful and the patient was discharged on the seventh postoperative day.

Macroscopic assessment of the resected specimen showed the presence of a giant (8.5 × 5 × 3 cm) colic polypoid lesion near the ileocecal valve, causing intussusception of a 12 cm long intestinal segment.

After the cut the polyp showed a yellow homogeneous nodule with well demarcated margins.

The final result was polypoid colonic lipoma causing intussusception and moderate ischemic damage with reactive lymphadenitis.

## 3. Discussion

Intussusception is a rare condition in adults (1% of bowel obstructions). 90% of cases have an organic cause, 60% due to neoplasm (60% malign and 40% benign) [2]; in particular 65–70% of adult colonic intussusceptions are caused by carcinomas [3]. Colonic lipoma is the most common benign tumor which causes colonic intussusception in adults, but very rarely [5].

Colonic lipomas are more common in women with a peak incidence between 50 and 60 years of age [6, 7]. They are mostly located in the right colon: 19% in cecum, 38% in ascending colon, 22% in transverse colon, 13% in the descending colon, and 8% into the sigma [4].

They arise from the submucosa in approximately 90% of cases, occasionally extending into the muscularis propria, and up to 10% are subserosal [8]. The size described in the literature ranges from 2 mm to 30 cm. They are multiple in 10–20% of cases and infrequently are pedunculated [4, 9, 10].

In general colonic lipomas are silent. Only 25% of patients develop symptoms: history of abdominal pain from mild to severe cramping followed by spontaneous improvement and recurrent episodes of constipation, nausea, and vomiting. Size of the lipoma is a predictor of symptomatology: lipomas larger than 4 cm cause symptoms in 75% of cases.

After intussusception abdominal pain is associated with vomiting, palpable mass, and bloody stool, presenting for many days or even weeks [3, 4, 7].

For the diagnosis, colonoscopy allows direct visualization of the submucosal lipoma, which appears as a mass covered by normal mucosa, but it can also show ulcerated or necrotic overlying mucosa [4, 8, 11]. Colonoscopic biopsy confirms the nature, but inadequate tissue samples often indicate nonspecific colitis with mucosal inflammation [12].

In case of intussusception, abdominal CT scanning is the radiologic modality of choice, above all when giant lipomas are present, with a 70–80% sensitivity and near 100% specificity [3, 13]. Lipoma appears with fat-equivalent density, near ovoidal shape, and smooth margins. However, intussuscepted lipomas may have a heterogeneous appearance reflecting the degree of infarction and fat necrosis [3, 14].

There are different options for treatment.

Small lipomas, less than 2 cm, can be endoscopically removed. Since lipomas show no malignant degeneration, if the biopsy is unequivocal, they may not need treatment and can be observed [4].

Some authors have reported that large pedunculated lesions can be removed without perforation using clipping or endoloop ligation [13, 15], but in most series endoscopic removal of lipomas larger than 2 cm is associated with a greater risk of perforation [4, 13].

It is therefore recommended that tumors larger than 2 cm must be resected surgically [13].

Moreover, the size of the lipoma is an essential factor leading to colonic intussusception, particularly when main axis of the lesion is over 4 cm. This is the reason why colonic lipomas of 4 cm or more must be resected before intussusception occurs [4].

The presence of intussusception leads to an emergency operation [3, 4].

Chiang recommended operative reduction for small bowel intussusceptions, but not for colonic ones [15].

If a colonic lipoma is diagnosed before surgery, segmental resection is an adequate treatment [4].

Since the majority of colonic intussusceptions are caused by primary adenocarcinoma, in view of the uncertain etiology, nondiagnosed lipoma before operation must be interpreted as for cancer, and a more or less extensive resection of the colon is recommended, depending on the location of the tumor. Patients must undergo more or less extensive resection of the colon also depending on the length of the intussusception segment [4, 15].

Even if additional cases are needed to optimize the standard management, surgery is always the treatment of choice and produces an excellent prognosis.
